# Evaluating ‘Enhancing Pragmatic Language skills for Young children with Social communication impairments’ (E-PLAYS): a feasibility cluster-randomised controlled trial

**DOI:** 10.1186/s40814-020-00724-9

**Published:** 2021-01-04

**Authors:** Suzanne Murphy, Victoria Joffe, Louisa Donald, Jessica Radley, Sailaa Sunthararajah, Charlie Welch, Kerry Bell, David Messer, Sarah Crafter, Caroline Fairhurst, Belen Corbacho, Sara Rodgers, David Torgerson

**Affiliations:** 1 Institute of Health Research, University of Bedfordshire, University Square, Luton, LU1 3JU UK; 2 University of Essex, Wivenhoe Park, Colchester, CO4 3SQ UK; 3 Department of Psychiatry, University of Oxford, Warneford Hospital, Oxford, OX3 7JX UK; 4 Research and Development Office, North East London NHS Foundation Trust, Goodmayes Hospital, Barley Lane, Ilford, IG3 8XJ UK; 5 Department of Health Sciences, University of York, Heslington, YO10 5DD UK; 6 Education & Language Studies, Faculty of Wellbeing, Open University, Walton Hall, Kents Hill, Milton Keynes, MK7 6AA UK; 7 School of Psychology, Faculty of Arts & Social Sciences, Open University, Walton Hall, Kents Hill, Milton Keynes, MK7 6AA UK

**Keywords:** Social communication, Pragmatic language, Randomised controlled trial, Feasibility study, Young children, Peer collaboration, Communication impairment, Computer game

## Abstract

**Background:**

This article reports the results from a feasibility study of an intervention (‘E-PLAYS’) aimed at supporting children who experience difficulties with social communication. E-PLAYS is based around a dyadic computer game, which aims to develop collaborative and communication skills. A pilot study found that when E-PLAYS was delivered by researchers, improvements on communication test scores and on collaborative behaviours were observed. The aim of this study was to ascertain the feasibility of running a full-scale trial to test the effectiveness of E-PLAYS in a National Health Service (NHS) setting with delivery by speech and language therapists and teaching assistants.

**Methods:**

The study was a two-arm feasibility cluster-randomised controlled trial of the E-PLAYS intervention with a treatment as usual control arm. Data relating to recruitment and retention, treatment fidelity, acceptability to participants, suitability of outcomes and feasibility of collecting health economic measures and of determining cost-effectiveness were collected.

Speech and language therapists selected suitable children (ages 4–7 years old) from their caseload. E-PLAYS intervention (experimental group) was then delivered by teaching assistants overseen by speech and language therapists. The control group received usual care. Assessments included blinded language measures and observations, non-blinded teacher-reported measures of peer relations and classroom behaviour and non-blinded parent-reported use of health and education resources and quality of life.

**Results:**

Planned recruitment was for 70 children, in the event, 50 children were recruited which was sufficient for feasibility purposes. E-PLAYS was very highly rated by children, teaching assistants and speech and language therapists and treatment fidelity did not pose any issues. We were able to collect health economic data which suggests that E-PLAYS would be a low-cost intervention.

**Conclusion:**

Based on recruitment, retention and adherence rates and our outcome measures, a full-scale randomised controlled trial estimated appears feasible and warranted to assess the effectiveness of E-PLAYS for use by the NHS and schools.

**Trial registration:**

ISRCTN 14818949 (retrospectively registered).

## Key messages

- What uncertainties existed regarding the feasibility? The study aimed to establish the feasibility of delivering the E-PLAYS intervention in schools. An earlier pilot study by the authors had demonstrated that, when delivered by researchers, an earlier version of E-PLAYS produced significant gains in pragmatic language test scores and communication skills compared to a control group. The areas of uncertainty concerned delivery of E-PLAYS by speech and language therapists and teaching assistants and the impact that this may have on intervention fidelity and acceptability, also the ease of recruitment for a large-scale study.
- What are the key feasibility findings? Acceptability was very high and intervention fidelity was good. However, there were some challenges in relation to recruitment and retention. Recruitment was via speech and language therapists who examined their caseloads for children fitting the inclusion and exclusion criteria and then approached schools. One unintended consequence of this recruitment strategy was that the demographic profiles of the parents of the children recruited were largely White British, graduates and in fulltime employment. This was not reflective of the North East London area in which we were recruiting which was ethnically highly-diverse and where several boroughs experience high levels of poverty. However, this was consistent with previous research which has found that well-educated parents with higher socio-economic status are better able to access services such as speech and language therapy.
- What are the key implications of the feasibility findings for the main study? Based on the outcomes of our study, a full-scale trial appears feasible and warranted to assess the effectiveness of E-PLAYS for use by the NHS and schools. The low-cost, computer-based nature of E-PLAYS makes it highly suitable for national distribution. E-PLAYS was designed ultimately to be widely shared and we have used existing, easily updated technology which is available within the NHS and in primary schools. A vast number of commercially-available games are targeted at parents of children with autism spectrum disorders and other children with social communication impairments. Reviewers of the computer game literature have urgently called for large-scale studies to ensure these are based on a sound evidence-base. A possible recruitment alternative would be to approach schools directly without using speech and language services as an intermediary. It is likely that this would reach a wider demographic in terms of socio-economic status and ethnicity.

## Background

Children with social communication impairments struggle to communicate effectively in social contexts; our E-PLAYS intervention aims to provide them with support. Difficulties with social communication can manifest themselves as an inability to maintain a topic of conversation or take turns appropriately, misunderstanding of non-literal language such as jokes, irony or sarcasm, failure to make inferences and repair communication breakdowns. Responding appropriately, interpreting others’ communications whilst at the same time understanding society’s norms and expectations constitutes pragmatic language skill [1, 2].

The terms ‘social communication’ and ‘pragmatic language’ are often used interchangeably. Recent systematic reviews of interventions for ‘social communication impairments’ or ‘pragmatic language impairments’ [3, 4] have adopted broad definitions for these terms encompassing both verbal and nonverbal aspects of communication to include abilities such as facial expression use as well as spoken language [5]. For the purposes of our study, however, we have adopted Matthews et al.’s (2018) [6] suggestion and defined ‘pragmatics’ more specifically as the *linguistic* component (excluding facial communication and other non-verbal communication) of social communication.

Children with social communication impairments are highly heterogeneous. The Diagnostic and Statistical Manual of Mental Disorders (DSM-V [7];) and the International Statistical Classification of Diseases and Related Health Problems (ICD 10 [8]) both list a number of developmental disorders (such as autism) and language disorders concerned with social communication. Furthermore, ADHD [9] and conduct disorders [10, 11] have also been shown to impact on social communication.

It is important to remember, however, that many children will experience difficulties with social communication without ever receiving diagnoses and there are indications that this is particularly the case for children of low socio-economic status [10, 12, 13].

Pragmatic language difficulties can have profound and long-lasting effects on emotional functioning and peer relations [14, 15]. Children are frequently rejected and victimised by peers [16, 17], and in adulthood, social relationships continue to be a challenge [18].

Schools aim to facilitate children’s learning and train team-building skills for adulthood through collaborative activities [19–22]. Collaborative working has also been shown to improve peer relations and facilitate children’s feelings of belonging [23, 24]. A systematic review by Chang and Locke (2016) [25] concluded that the inclusion of peers was one of the most promising strategies for social skills intervention. A recent review [26] concluded that collaborative learning activities are particularly beneficial for low-ability children at younger ages (4–7 years old).

Studies which have observed peer collaborations involving children with social communication impairments [27–29] have consistently shown that such children fail to contribute appropriately, sharing less, making more irrelevant statements, ignoring others and showing aggressive or withdrawn behaviours and consequently, they are often excluded by peers. Enabling social interaction between children with social communication impairments and their peers demands considerable skill from adult facilitators. The difficulties and resource implications of providing support for such children have tended to result in their isolation within the classroom; Blatchford et al. (2009) [30] have reported that typically, the most common activity for children with special educational needs is one-to-one working with teaching assistants and that consequently, rates of interaction with teachers and peers are reduced by almost half [31].

There are few interventions available to SLTs and schools for pragmatic language difficulties [3, 4, 32]; the Social Communication Intervention Programme (SCIP) is an example of an intervention with emerging evidence [33] but it demands a substantial time input from SLTs. The use of technology and gaming have been promoted as particularly useful means of facilitating communication and collaboration for children with communication difficulties as they are especially appealing to this group of children e.g. [34–36]. A systematic review and meta-analysis of computer-assisted collaborative learning [37] concluded that the intrinsic socio-cognitive scaffolding can enhance collaborative skills substantially; thus, computer games can facilitate communication and collaboration between children [38–40]. Alt et al. (2012) [41] have pointed out that currently, peer collaborative activities for children with communication and language impairments rely heavily on adult specialist skill for facilitation; little time or thought has been invested in the *creation and development* of these activities. Technology can fill this gap and be used to regulate turn-taking, participation and enforce the rules [42] and can also add surprises, colourful animations and unusual sounds and keep the game flowing at a suitable pace [35, 43].

Preliminary testing (*n* = 32) for ‘E-PLAYS’ (formerly known as the ‘Maze Game’) is described in Murphy et al. (2014a, 2014b) [29, 44]. Children in the intervention group showed significant increases on communication test scores by comparison to a control group [44]. In this preliminary study, the intervention was delivered to the children by the research team The aim of the present study was to establish the feasibility of running a full-scale clinical trial to evaluate E-PLAYS’ clinical- and cost-effectiveness when implemented within the NHS.

The study had the following feasibility objectives; to assess:

1. Participant recruitment and retention (via NHS trusts and schools)
2. The acceptability of E-PLAYS to children, SLTs, teachers, parents and teaching assistants
3. Treatment fidelity.
4. The suitability of outcome measures
5. The feasibility of collecting health economic measures and of determining cost-effectiveness for a full trial

## Method

### Feasibility trial design

The study was a two-arm, cluster-randomised trial (cRCT) to investigate the feasibility of conducting a sufficiently powered trial to evaluate the effectiveness and cost-effectiveness of E-PLAYS (compared with treatment as usual) for young children with social communication impairment.

### Setting

We recruited speech and language therapists (SLTs) employed by North East London NHS Foundation Trust (NELFT) Speech and Language Services.

### Participants

#### Speech and language therapists

The research team approached all suitably employed paediatric SLTs. They were invited to presentations by the team where they had the opportunity to ask questions and receive an information sheet and consent form to take away. Consent forms were returned by post, electronically or by hand to the research team. SLTs not responding within a week were reminded and invited again by email once more only.

#### Focal children

##### Inclusion criteria

SLTs screened all children on their caseloads and identified children aged 4 to 7 years who attended mainstream schools using the Social Communication Behaviour Checklist devised by Adams et al. (2012) [45]. Children were required to meet at least two of the five criteria as based on the SLT’s clinical judgement below:

- The child has trouble understanding and interpreting the social context and friendship, e.g. social roles, emotions
- The child has trouble understanding and/or using non-verbal aspects of communication, e.g. facial expression, intonation
- The child has trouble with aspects of conversation, e.g. beginning and ending, taking turns, giving relevant and sufficient information
- The child makes bizarre, tangential or inappropriate comments
- The child has difficulty using and understanding non-literal language

Children were also required to have at least minimum levels of English (children with English as an additional language were included). We wished to be as inclusive as possible; teachers were consulted about children’s level of English and all those regarded as having sufficient levels of English were included. The parents of every child meeting the above criteria were invited to participate in the study.

##### Exclusion criteria

- Hearing, visual or physical impairment severely affecting speech production

#### Parents

The research team provided schools with participant information sheets to send to parents of eligible children, one for parents and one written in simpler language for the child, with consent forms for parents to sign to authorise their child’s participation and to indicate their willingness to complete health economic questionnaires.

#### Teachers and teaching assistants

The teachers and teaching assistants of focal children were invited to take part in the study and received information sheets and consent form. Some teachers and teaching assistants were associated with more than one focal child.

#### Partners for focal children for the E-PLAYS intervention

Suitable partner children were suggested by teachers and teaching assistants in consultation with the SLT. These partner children were typically-developing children without language disorders in the same class as the focal children. Schools were provided with participant information sheets for parents of the partner children, again one for parents and one for children, with a consent form.

All participants were given the option of contacting the Chief Investigator in the event of additional questions and were informed that they were free to withdraw at any time from the study.

### Randomisation and allocation process

Focal children were cluster-randomised at the level of their treating SLT. The SLTs were randomised 1:1 to the intervention or comparator group after they had consented to participate in the study and had identified and recruited children on their caseloads but before they received a briefing on E-PLAYS delivery from the research team. Allocation was via minimisation to ensure balance across the two groups on the borough of the SLT (five boroughs covered by NELFT, each served by a different SLT team) and the number of children recruited (dichotomised around the median). Minimisation was implemented by a trial statistician at York Trials Unit using MinimPy version 0.3.

### Blinding

Due to the nature of the intervention, SLTs, teachers, teaching assistants, children and parents could not be blinded to allocation. However, the research assistants collecting outcome data were blinded (see the ‘Measures’ section). Several different research assistants were associated with this project and they did not conduct pre- and post-test measures on the same children. We also ensured that same research assistants did not conduct qualitative assessments (where they were unblinded) with the same children to whom they administered the TPS, CCT and Recalling Sentences tests. At each visit to schools, staff were reminded not to unblind research assistants.

### Procedure and intervention

#### Intervention group: E-PLAYS

E-PLAYS has been designed to be administered by non-specialists such as teaching assistants (as opposed to SLTs or teachers) in 12 weekly sessions. SLTs randomised to the intervention group received a 30-min briefing from the research team together with a manual. The SLTs then trained the teaching assistants in small groups for around 2 h in their schools and gave them the manual with which to deliver E-PLAYS. SLTs gave teaching assistants further support in the same way as they would usually when introducing a new intervention if needed. Generally, most teaching assistants were able to use the game readily, the support that was given was very brief and amounted to a maximum of 30 min per teaching assistant.

The E-PLAYS intervention is built around a dyadic collaborative computer game. Children play the computer game with a teaching assistant for eight, 30-min sessions and with a classmate for four, 15-min sessions. In their sessions, teaching assistants use the game to guide the child through (a) requesting optimally useful information, (b) giving helpful directions and answering questions and (c) asking for clarification. Sessions with the classmate give the focal child an opportunity to practice in live interaction the content of the sessions led by the teaching assistants. Sessions one, five, six and twelve were those with a peer; the other sessions were with the TA only. Session one was designed as an introductory session for the children to find their way around E-PLAYS, sessions five and six were in the middle, for children to practice new skills and the final session, twelve, aimed to consolidate the skills learned.

#### Comparator group: usual practice

Usual practice for a child with social communication impairment on an SLT’s caseload typically comprises a programme of activities, devised by the SLT who supports schools’ teaching assistants to deliver them (a ‘consultancy model’ [46]). Activities typically [32, 47] include exercises on turn-taking, topic management and conversational skills, sometimes with role-playing or modelling. These are generally taught directly by an adult (e.g. a teaching assistant) to the child one-to-one or in a small group.

For both the intervention and the comparator group, SLT visits to support the teaching assistants took place around 3–6 times per year.

### Measures

#### (a) Measures administered and rated by blinded research assistants

##### (i) Test of Pragmatic Skills, (TPS, [48])

A major difficulty inherent in researching pragmatic language skills is that social communication difficulties generally manifest only during social interaction itself and are therefore usually missed by standard language tests [1]. Social communication impairments are therefore best assessed by direct observation. The TPS is an observational elicitation measure. The tester engages the child in structured but naturalistic play to elicit target behaviours and responses, which are audio-recorded and later scored. Scoring time for the TPS is approximately 25 min. The TPS was a sensitive indicator and successful at detecting improvements in communication in our pilot study [44]; it has been standardised on 650+ children by the author. The instrument shows good reliability (test-retest *r* = 0.96, interrater *r* = 0.92). A score between 0 and 42.5 is given, where a higher score indicates greater pragmatic language skill.

##### (ii) Clinical Evaluation of Language Fundamentals-5 (CELF-5, [49]).

CELF-5 is one of the most widely-used standard language tests by SLTs. We used one of the subscales only, Recalling Sentences. This sentence repetition assessment is generally regarded as a measure of overall language ability drawing upon a wide range of language processing skills [50]. The type of language structures that are modelled in the intervention (e.g. requesting clarification, providing directions) could potentially lead to greater syntactic competence and an ability to structure more coherent sentences. This subscale therefore gives us an indication of the impact of E-PLAYS on children’s language as a whole by comparison to the more specific social communication skills targeted by the TPS.

Raw scores (0–78) are used to calculate age-adjusted scores between 1 and 19, where higher scores indicate greater ability to recall and reproduce spoken sentences accurately.

##### (iii) Dyadic collaborative construction task (CCT)

A frequently reported issue with interventions targeting children with social communication impairments is that the skills learned do not generalise beyond those of the intervention context; furthermore, this skill transfer is rarely measured [4]. A 10-min, collaborative construction task using Magformers® (a construction toy made with plastic, brightly coloured, magnetised blocks) was devised by the team as a transfer measure to observe children’s collaborative and communicative skills pre- and post-intervention. Children undertook the task with a peer (this was the same classmate pre- and post-test and also for the intervention) and were video-recorded by research assistants. The children followed instructions and were asked to work together without adult input. What we were observing, in this instance, was the ability, not only of the peer to scaffold the focal child, but also of the focal child to build up a collaborative working relationship with the peer over time.

#### (b) Teacher-completed measures

##### (i) The Children’s Communication Checklist-2 (CCC-2, [51])

The CCC-2 is the most widely used, standardised questionnaire of communication impairment in research and clinical contexts [1]. The CCC-2 has eight subscales which can be combined to yield the General Communication Composite, an indicator of overall communication difficulty, on a scale from 3 to 133 (age-dependent; higher scores indicate fewer difficulties). It also includes four subscales (Initiation, Stereotyped Language, Use of Context and Non-verbal Communication) that may be considered to be more specifically concerned with pragmatic language and can be combined to give a Pragmatic Language Score [6] between 3 and 69 (age-dependent, higher scores indicate fewer difficulties).

##### (ii) The Strengths and Difficulties Questionnaire (SDQ, [52]).

The SDQ is a widely used mental health indicator with subscales assessing behavioural, emotional and peer problems. A total score for the SDQ was calculated from 0 to 40 (higher scores indicate greater difficulties) and for the Prosocial subscale from 0 to 10 (higher scores indicate greater prosocial behaviour).

#### (c) Parent-completed measures

##### (i) Quality of Life: EQ-5D-Y (proxy 1) [53] and Paediatric Quality of Life (PedsQL, [54])

The EQ-5D-Y (proxy 1) was developed as a child-friendly version of the EQ-5D, the preferred instrument recommend by NICE for clinical decision-making. The proxy version asks caregiver to rate the child’s health-related quality of life. Given the population of the study, we were uncertain as to whether the EQ-5D-Y (proxy-1) could offer sufficient sensitivity, thus we also included the PedsQL as a comparative instrument.

##### (ii) Resource use

The ability to assess resource implications was piloted using a bespoke resource use questionnaire for parents. The costing approach was undertaken from an NHS perspective and also considered the perspectives of both Social Services and education providers.

#### (d) Fidelity measures

Delivery of the computer game within E-PLAYS (duration and number of sessions) was automatically recorded by the E-PLAYS software and transmitted to the research team; each child could be identified individually by their unique PIN login.

### Process evaluation – qualitative investigation

A process evaluation was included in our study (as recommended by the Medical Research Council (MRC) guidance for RCTs, [55]) to elucidate processes which may impact on intervention delivery and measurement. Processes examined were whether:

- Instructions for delivery of E-PLAYS were considered to be adequate
- Staff could use E-PLAYS with sufficient fidelity
- How acceptable staff and children found E-PLAYS

SLTs and teaching assistants in the intervention arm received open-ended questionnaires within 2 weeks of receiving the manual (SLTs) and of training (teaching assistants); these assessed the clarity of instructions for delivery of E-PLAYS. Ten teaching assistants in the intervention group were invited to be observed whilst delivering E-PLAYS and together with the corresponding children were viewed live by research team members for two E-PLAYS sessions each, observation of these sessions concerned teaching assistants’ adherence to manual instructions and teaching assistants were made aware of this. These same children were also interviewed about their experiences of E-PLAYS. Focus groups with teaching assistants were conducted at the end of the summer term in which E-PLAYS was delivered. The child interviews and focus groups were concerned with acceptability.

### Data collection points for measures and qualitative assessments

Data collection for the TPS, CCC-2, CELF-5 and SDQ took place at baseline, 15–20 weeks and 35–40 weeks post-randomisation. These time points are approximately equivalent to baseline, immediate post-intervention and 3-month post-intervention follow-up for the intervention group.

Analysis of the CCT is a resource-intensive research activity requiring substantial input of research assistant time. Therefore, as this was a feasibility study, the decision was taken to collect at baseline and 15–20 weeks only to save resources as the study was primarily concerned with the viability and acceptability of collecting this data rather than producing definitive analyses. All health economic measures and demographic data were collected at 35–40 weeks post-randomisation. These data were collected at one time point only as we have observed in previous trials that whilst baseline data collection tends to be good, completion rates decline over time hence administering the questionnaire at the end time point gives us an indication of the likely minimum level of data attainable whilst also reducing participant burden.

### Sample size

We aimed to recruit 70 focal children to provide reliable estimates of sample size parameters [56].

### Analysis

Analyses were conducted in Stata v15. Following CONSORT recommendations [57] for feasibility studies, quantitative outcome measures were summarised descriptively by group and time point, with no formal between-group comparisons undertaken. Parameters required to calculate the sample size for a future full-scale RCT were estimated. The variance of the TPS at each time point was calculated with a 95% confidence interval (CI). To estimate the intra-cluster correlation coefficient for TPS scores at week 20 and 40, we used a three-level mixed effects model (measurements nested in children nested in SLTs), adjusting for the main effects of time and allocation and their interaction, baseline TPS score and the factors used in the minimisation algorithm as fixed effects with random intercepts for SLT and child nested in SLT. The correlation and 95% CI between the TPS score at baseline and the two post-randomisation time points were calculated.

The focus groups were audio-recorded and professionally transcribed. Observations of the teaching assistants were live and followed a checklist. Thematic analysis for both followed the guidelines of Braun and Clarke (2006) [58]: (1) becoming familiar with the data, (2) generating initial codes, (3) searching for themes, (4) reviewing themes and (5) defining and naming themes.

Data from the open-ended questionnaires were analysed by coding responses into categories known as a ‘coding frame’ [59]. Children’s interviews were conducted using the Fun Toolkit [60], a schedule designed to probe children’s views of technology. The Fun Tool Kit asks children to rank the aspects of the game that they liked best and to give an overall indication of how much they liked the game.

For the CCT, video-recordings were transcribed by a professional company and then coded by blinded research assistants. Coding consisted of observation and categorisation of communicative (e.g. Information-Seeking Questions, Directives, Clarification Requests) and affective (e.g. positive and negative behaviours) items which have been found to differentiate children with social communication impairments from typically-developing children [44]. The coding system is based on micro-analytic theory [61, 62] and was developed by the team specifically to analyse children’s collaborative interaction. It was based on research in collaborative learning, conversation analysis and language impairment [29, 44, 63–69]. We have used this system extensively and it has previously successfully detected changes in talk in our studies [29, 44, 64]. Research assistants were trained to fidelity by the Chief Investigator by watching and coding training videos until they achieved a threshold of concordance (that is, agreement with 80% of the codes on the training videos). Inter-rater reliability was also calculated between research assistants taking part in this study using a random sample of 15% of all video-recordings.

The costs of providing E-PLAYS were estimated including accounting for development and ongoing maintenance costs where possible. An estimate of the costs of running E-PLAYS within the NHS and schools was calculated.

## Results

### Participant recruitment and flow

**Fig. 1:**
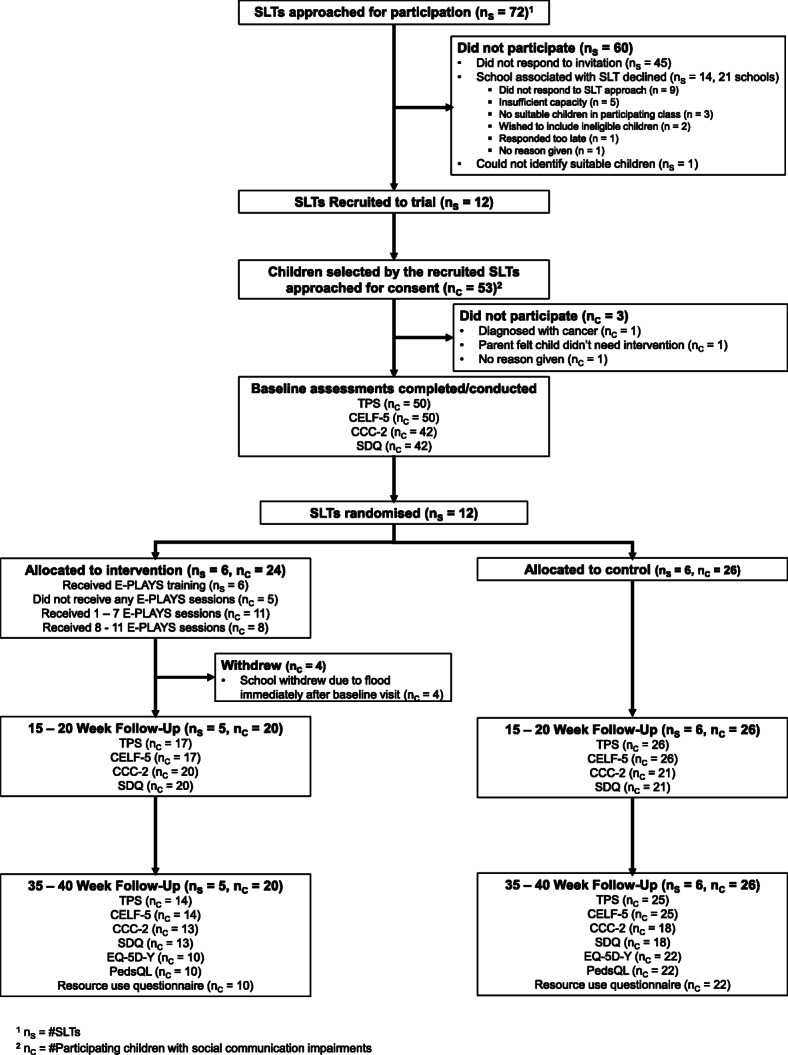
Study flow diagram

We approached 72 SLTs for participation, of which 45 (63%) did not respond, 14 (19%) were associated with a school which declined to participate and one (1%) could not identify any potentially eligible children on their caseload (Fig. 1). The remaining 12 SLTs (17%) were randomised into the trial (six to each group). Recruitment of focal children commenced on 1st January 2018 and closed on 20th April 2018. Recruitment of children (via parents) did not take place until schools had agreed to participate. Recruitment was originally planned for September 2017–December 2017 but had to be delayed. We recruited 50 focal children out of a target sample size of 70 (24 in the intervention group and 26 in the control). These children were recruited from 14 schools (six intervention and eight control). One school (four children) withdrew immediately following the baseline visits due to flooding at the school. Another school (six children) was unresponsive at the final data collection point meaning no assessments could be undertaken. SLTs recruited a mean of 4.2 children (SD 1.7, range 2 to 7). Children were aged, on average, 6.2 years (0.8) and 74% were male (Table 1). Characteristics relating to age, gender and baseline assessments conducted appear comparable between the two groups.

**Table 1:** Characteristics of participants for intervention (E-PLAYS) and comparator groups

	**E-PLAYS (*n*** **= 24)**	**Comparator (*n*** **= 26)**
**Age at study entry (years)**		
Mean (SD)	6.2 (0.8)	6.3 (0.8)
**Gender,** ***n*** **(%)**		
Male	17 (70.8)	20 (76.9)
Female	7 (29.2)	6 (23.1)
Missing	0 (0.0)	0 (0.0)
	**Intervention (*n*** **= 10**^**a**^**)**	**Comparator (*n*** **= 22**^**a**^**)**
**Ethnicity,** ***n*** **(%)**		
White British	5 (50.0)	9 (40.9)
White other European	0 (0.0)	4 (18.2)
White other	1 (10.0)	1 (4.6)
Black African	1 (10.0)	1 (4.6)
Black Caribbean	0 (0.0)	1 (4.6)
Indian	1 (10.0)	2 (9.1)
Pakistani	0 (0.0)	2 (9.1)
Bangladeshi	1 (10.0)	0 (0.0)
White and Black Caribbean	0 (0.0)	2 (9.1)
Missing	1 (10.0)	0 (0.0)
**Parent qualifications,** ***n*** **(%)**		
No formal qualifications	1 (10.0)	0 (0.0)
Some qualifications	2 (22.0)	7 (31.8)
Degree or higher	4 (40.0)	13 (59.1)
Prefer not to say	2 (20.0)	2 (9.1)
Missing	1 (10.0)	0 (0.0)
**Parent employment,** ***n*** **(%)**		
Employed full time	4 (40.0)	6 (27.3)
Employed part time	1 (10.0)	3 (13.6)
Self-employed (full time)	0 (0.0)	2 (9.1)
Self-employed (part time)	0 (0.0)	1 (4.6)
Not in paid work	1 (10.0)	5 (22.7)
Student	1 (10.0)	1 (4.6)
Other	0 (0.0)	2 (9.1)
Prefer not to say	2 (20.0)	2 (9.1)
Missing	1 (10.0)	0 (0.0)
**Parent relationship,** ***n*** **(%)**		
Single	1 (10.0)	2 (9.1)
Married/cohabiting	6 (60.0)	14 (63.6)
Divorced	1 (10.0)	3 (13.6)
Separated	1 (10.0)	1 (4.6)
Prefer not to say	0 (0.0)	1 (4.6)
Missing	1 (10.0)	1 (4.6)

^a^Age and gender collected at baseline, other demographic data collected at 35–40-week follow-up

Fifty typically-developing children were recruited to partner the focal children; 100% of parents (*n* = 50) gave consent for these children to take part in the study. All participating SLTs (*n* = 12) and 35 out of 39 participating teaching assistants returned the open-ended questionnaires. There were 19 teaching assistants in the intervention group, 14 of these participated in the focus groups which was as many as were available on the days in question, all had been invited. Our aim was to observe ten teaching assistants; ten were randomly selected and approached. Of these, one declined, and therefore, nine teaching assistants were observed delivering two E-PLAYS sessions and nine children were observed in the same E-PLAYS sessions and were then interviewed by research assistants using the Fun Tool Kit [60].

The response rates for research assistant-administered measures were 86% at 20 weeks and 78% at 40 weeks. For teacher-completed measures response rates were 82% at 20 weeks and 62% at 40 weeks. Forty parent questionnaires were distributed at 40 weeks, of which 32 were returned.

### Acceptability

#### Speech and language therapists

Responses to the questionnaire from SLTs indicated that the training manual was favourably regarded by SLTs and they commented on its clarity:

I found the manual very clear (SLT 2)

…the manual is so self-explanatory(SLT 5)

…the session by session breakdown [is] very helpful (SLT 12)

#### Teaching assistants

Similarly, teaching assistants commented positively on the manual and especially on the illustrations:

Simply written. Easy with diagrams (TA4)

When it was unclear, I looked at the pictures and it helped me (TA7)

Step by step explanations also very easy to follow (TA10)

There were few problems with running the game itself, which had been designed to be easy to load and use. During focus groups, teaching assistants were overwhelmingly enthusiastic about the game.

He (name of child receiving intervention) loved it… he really did love it. (TA4)

The game is brilliant (TA8)

It does make it fun that it is a game and it does help them and make them want to

do it (TA9)

Teaching assistants were especially positive about the involvement of typically-developing partner children and stressed that both the partners and the focal children enjoyed the game.

I think they are more relaxed with peers than an adult (TA2)

…he really listened well; he listened better to (partner name) than he does in class. (TA8)

#### Children

Children appeared to like all aspects of the game, particularly finding the ‘hidden treasures’. Six of the nine children interviewed rated the game as ‘good’, ‘really good’ or ‘brilliant’. Teaching assistants explained that one of the children who rated the game as ‘not very good’ was upset at having to finish (because of school timetabling) the game sooner than they wanted and the two others were ‘having a bad day’ but did in general at other times enjoy E-PLAYS.

#### School use

A further indication of the acceptability and popularity of E-PLAYS is that after completion of data collection, control group schools were offered the use of E-PLAYS. All control group schools took up this offer; furthermore, many of the intervention group schools asked to continue using E-PLAYS, with the result that we extended the licence to all participating schools until December 2019. In total, nine of the 14 participating schools (2 of 6 in the intervention and 7 of 8 in the control group) were still using E-PLAYS 12 months after study completion.

### Intervention delivery and fidelity

#### Intervention fidelity: qualitative observations

During observations of the teaching assistants, we found that all were able to use the software and set up the game and all were able to keep the children on task. Less positively, only three of the observed teaching assistants followed manual recommendations to pause to give the children time to respond to questions and remind children of the collaborative nature of the task. Almost all teaching assistants tended to intervene with the children more than recommended instead of allowing the children to explore the game.

The unanticipated delays in recruitment had a detrimental, knock-on effect on intervention delivery. E-PLAYS is a 12-session intervention with one (recommended) session per week. Due to the recruitment delays, many schools were left with only the end of the summer term in which to deliver 12 sessions of E-PLAYS and this was often less than 12 weeks. Schools were therefore asked to deliver as many sessions as was reasonably practicable. The mean number of sessions received by the children in the intervention group was 6.2 (SD 4.3). Five children randomised to the intervention group did not receive any E-PLAYS sessions. This was due to one school being unable to commence E-PLAYS for one child and another school (four children) withdrawing immediately following the baseline visit due to flooding at the school. Excluding the children from the school which withdrew, then the mean number of sessions received was 7.4 (SD 3.6). However, once schools were able to begin delivering E-PLAYS, the average frequency with which they delivered the E-PLAYS sessions was within the recommended range. The mean frequency of session completion was 1.1 sessions per week (range 0.3–2.6). Recommended duration for the sessions where the focal child played with the teaching assistant was 30 min (minimum 20 min, no maximum); actual mean duration was 25.50 (SD 11.6) min. Recommended duration for child and classmate peer partner sessions was 15 min (minimum 10 min, no maximum); actual mean duration was 28.72 (SD 12.47) min.

Children in the intervention group generally received the early sessions with declining numbers receiving the later sessions; all 19 children received session 1 but only six children received session 12. Peer sessions required as much adult input (in terms of supervision) as did the teaching assistant only sessions so this was not a factor impacting delivery. To illustrate, the number of children receiving session 4 (teaching assistant only) = 17, session 5 (peer session) = 16, session 6 (peer session) = 12 and session 7 (teaching assistant only) = 11 and session 12 (peer session) = 6.

### Suitability of outcome measures and qualitative processes

From the point of view of participant burden and ease of administration, these outcome measures were generally found to be suitable. Importantly, the TPS, CELF-5 and CCT were blinded independent measures and we were able to maintain blinding. On two occasions school staff did inadvertently unblind research assistants despite being reminded. On these occasions, a different (blinded) research assistant was substituted to administer the outcome measure.

Table 2 gives scores for research assistant-administered and teacher-report measures. Improvements in TPS score over time were observed in both groups, as might be expected for this population. Scores for other outcome measures were more mixed. There is generally limited evidence for large differences between groups; few conclusions can be drawn, due to the small number of participants and the possibility of informatively missing (missing not at random) outcome data.

**Table 2:** Scores for measures for the intervention (E-PLAYS) group and for the comparator group at baseline, 20 weeks post-randomisation and 40 weeks post-randomisation

	E-PLAYS (***N*** = 24)			Comparator (***N*** = 26)		
	Baseline	20 weeks	40 weeks	Baseline	20 weeks	40 weeks
**Research assistant completed**						
**TPS**^a^						
N	23	16	14	26	26	25
Mean (SD)	23.6 (5.6)	23.7 (6.6)	26.2 (7.2)	21.9 (8.3)	23.8 (7.4)	25.7 (6.9)
**CELF-5**^b^						
N	24	17	14	26	26	25
Mean (SD)	6.2 (2.8)	7.2 (2.5)	5.8 (2.5)	5.5 (3.1)	6.5 (3.0)	6.7 (2.9)
**Teacher completed**						
**SDQ (total difficulties)**^c^						
N	20	19	13	22	21	18
Mean (SD)	18.3 (6.4)	15.9 (4.7)	19.5 (6.6)	16.4 (5.3)	15.2 (5.8)	14.1 (4.6)
**SDQ (prosocial)**^d^						
N	20	19	13	22	21	18
Mean (SD)	4.3 (2.6)	4.7 (2.4)	5.8 (2.1)	4.2 (2.7)	5.1 (2.2)	5.1 (1.7)
**CCC-2 (GCC)**^e^						
N	19	20	13	22	21	18
Mean (SD)	35.2 (11.2)	38.0 (15.8)	30.5 (11.8)	36.1 (14.6)	39.0 (19.1)	43.1 (13.8)
**CCC-2 (PLS)**^f^						
N	19	20	13	22	21	18
Mean (SD)	20.6 (6.8)	20.4 (7.3)	18.0 (7.0)	18.7 (5.3)	19.4 (7.7)	22.1 (6.4)

For all measures, higher scores indicate better outcomes except for SDQ (total difficulties) where a higher score indicates more difficulties

^a^Test of Pragmatic Skills

^b^Clinical Evaluation of Language Fundamentals-5; subscale Recalling Sentences only

^c^Strengths and Difficulties Questionnaire, total of all subscales except Prosocial

^d^Strengths and Difficulties questionnaire, Prosocial subscale only

^e^Children’s Communication Checklist, General Communication Composite

^f^Children’s Communication Checklist, Pragmatic Language Score, sum of (E—initiation; F—stereotyped language; G—use of context; and H—non-verbal communication subscales)

Scores in general are typical of children with language disorders. Of the 49 children with TPS scores at baseline, 45 (91.8%) had scores below the 25th percentile of the standardisation sample used in the development of the TPS and 26 (53.1%) had scores below the 10th percentile. Similarly, 48 (96%) of the children in the sample had baseline scores on the CELF-5 Recalling Sentences subscale either at or below the mean score obtained by the standardisation sample, with 34 (68%) having scores more than one standard deviation below the standardisation sample mean score. This was also reflected in the teacher-completed instruments. Of the 41 children with a valid baseline score for the CCC-2 General Communication Composite, all of them had scores below the 20th percentile of the standardisation sample, with 37 (90.2%) of these having scores below the 10th percentile. Of the 42 children with a valid SDQ Prosocial scale score at baseline, 35 (83.3%) had scores below the mean score for British children aged 5–10 years (mean = 7.3, SD = 2.4) and 23 (54.8%) had scores more than one standard deviation below this mean.

We successfully achieved inter-rater reliability between the research assistants (weighted kappa = 0.69). According to guidelines proposed by Landis and Koch (1977) [70], kappa values ranging from 0.41 to 0.60 are rated ‘moderate’, 0.61–0.80 as ‘substantial’ and 0.81–1 as ‘almost perfect agreement’. Table 3 shows scores of E-PLAYS and comparator groups for communication codes. Directives, Clarifications and Information Questions all showed increases over time. For Positive and Negative Feelings, results varied with no consistent pattern. Overall, no firm conclusions could be drawn especially as for this small sample, the impact of the time taken for the task cannot be controlled for.

**Table 3:** Collaborative Construction Task; number of codes appearing in each task for each pair of children for the intervention (E-PLAYS) group and for the comparator group at baseline, 20 weeks post-randomisation and 40 weeks post-randomisation

	E-PLAYS		Comparator	
	Baseline (***n*** = 24)	20 weeks (***n*** = 17)	Baseline (***n*** = 26)	20 weeks (***n*** = 26)
**Directives**				
Mean (SD)	3.3 (3.8)	4.5 (7.8)	4.3 (4.7)	6.3 (5.6)
**Clarification requests**				
Mean (SD)	1.5 (2.0)	3.0 (4.8)	1.4 (2.0)	2.6 (4.0)
**Information-seeking questions**				
Mean (SD)	4.6 (3.7)	5.0 (7.6)	3.9 (4.0)	4.6 (4.8)
**Positive feelings**				
Mean (SD)	3.1 (3.5)	1.8 (2.0)	3.0 (3.4)	2.3 (2.5)
**Negative feelings**				
Mean (SD)	3.2 (4.1)	2.6 (4.1)	1.2 (1.2)	2.1 (4.6)
**Task time (minutes)**				
Mean (SD)	11.3 (4.0)	14.1 (3.7)	11.2 (3.6)	12.7 (4.7)

### Cost and resource use data collection

#### (i) Quality of Life

Table 4 gives responses for EQ-5D (proxy-1) and PedsQL questionnaires. Due to the absence of published population norms for EQ-5D for children, we cannot compare how the reported levels of problems relate to those observed in the general population. However, only 35% of caregivers reported children being in a perfect health state with caregivers reporting various levels of issues across the 5 dimensions. The established national norm for the PedsQL of a healthy population is 82.3 (± 15.6) [54]. Based on the combined results of all responding caregivers, the children in the study score at the lower end of this normal range suggesting that the instrument is sensitive to the problems faced by these children.

**Table 4:** Proportions of reported problems by the trial arm in EQ-5D-Y dimensions and PedsQL scores

	E-PLAYS (***n*** = 10)	Comparator (***n*** = 22)
**EQ-5D-Y dimensions**		
**Mobility (walking about),** ***n*** **(%)**		
No problems	10 (100)	20 (90.9)
Some problems	0 (0.0)	2 (9.1)
A lot of problems	0 (0.0)	0 (0.0)
Missing	0 (0.0)	0 (0.0)
**Looking after him/herself,** ***n*** **(%)**		
No problems	4 (40.0)	8 (36.4)
Some problems	4 (40.0)	12 (54.5)
A lot of problems	1 (10.0)	2 (9.1)
Missing	1 (10.0)	0 (0.0)
**Doing usual activities,** ***n*** **(%)**		
No problems	5 (50.0)	12 (54.5)
Some problems	3 (30.0)	9 (40.1)
A lot of problems	1 (10.0)	1 (5.5)
Missing	1 (10.0)	0 (0.0)
**Having pain or discomfort,** ***n*** **(%)**		
No problems	9 (90.0)	19 (86.4)
Some problems	0 (0.0)	3 (13.6)
A lot of problems	0 (0.0)	0 (0.0)
Missing	1 (10.0)	0 (0.0)
**Feeling worried, sad or unhappy,** ***n*** **(%)**		
No problems	5 (50.0)	13 (59.1)
Some problems	3 (30.0)	8 (36.4)
A lot of problems	1 (10.0)	1 (5.5)
Missing	1 (10.0)	0 (0.0)
**EQ-5D visual analogue scale**		
*N*	9	19
Mean (SD)	83.1 (22.3)	86.7 (12.1)
**PedsQL scales**	**Mean (SD)**	**Mean (SD)**
Physical health	77.4 (23.3)	78.3 (19.0)
Psychosocial health	59.0 (25.0)	61.8 (18.2)
Emotional functioning	66.9 (27.6)	61.8 (23.2)
Social functioning	60.5 (22.3)	60.5 (27.0)
School functioning	63.0 (19.9)	71.1 (14.0)
Total scale	65.4 (23.6)	70.2 (18.3)

By comparison to the EQ-5D-Y (proxy-1), the PedsQL provides more in-depth information concerning emotional, social and school functioning, all dimensions that are likely to be affected by social communication impairments.

#### (ii) Resource use

Little NHS, Social Services and educational services use was reported across both trial arms either through the NHS or privately. Similarly, low levels of use were reported for home-based assistance, voluntary services or education and childcare services. The most widely used resource was special educational needs coordinator (SENCO) though the mean number of contacts was still very low: 2 (SD 3.5) in the comparator group and less than 1 (SD 0.5) in the E-PLAYS intervention group.

#### (iii) Intervention delivery: costing exercise

From an NHS perspective, low input is required from health care professionals to deliver E-PLAYS. We anticipate, therefore, the cost to the NHS to be small meaning if the intervention was effective it would likely represent good value for money. Schools are not required to purchase or provide any additional resources to facilitate the delivery of E-PLAYS. There was some time required to set up the intervention initially in schools and attend the training. Teaching assistants spent around 10 min preparing for each session. Schools participating in the feasibility study were not required to pay for the E-PLAYS software; however, in a full-scale trial, it is possible that schools would incur an annual licencing fee of approximately £50 per school to cover software maintenance costs. As with the NHS, costs for schools would be low.

### Estimating the sample size of a future trial to evaluate the effectiveness of E-PLAYS

The variance of the available TPS scores at baseline, week 20 and week 40 was 51.3 (95% CI^1^ 29.3 to 93.9), 49.1 (95% CI^1^ 30.0 to 80.6) and 47.8 (95% CI^1^ 23.5 to 93.3), respectively. These suggest a reasonable estimate of the standard deviation of 7.0 (although the available data are compatible with values between approximately 4.8 and 9.7). Conditional on the fixed effects, the intra-cluster correlation for SLT was essentially 0. In order to be conservative, we assume an intra-cluster correlation of 0.05 in the following calculation which, together with a (conservative) estimate of the mean cluster size of 5, gives an estimated design effect due to clustering of 1.20. The observed correlations between the baseline TPS score and the TPS scores at week 20 and week 40 were strong at 0.84 (95% CI^2^ 0.71 to 0.91) and 0.79 (95% CI^2^ 0.63 to 0.89), respectively. We can account for a more conservative correlation of 0.6 in our sample size calculation by multiplying the sample size required for an unadjusted analysis by(1 − 0.6^2^). Finally, we assume a conservative rate of attrition of 25%. These parameters mean that 356 children would be required for 90% power in a two-sided test of size 5%. Assuming an average of 5 children per SLT, approximately 71 NHS SLTs would need to be recruited and randomised.

### Adverse events and safety

No adverse events were recorded.

## Discussion

The aim of the present study was to assess the feasibility of conducting a full-scale randomised controlled trial to evaluate the effectiveness and cost-effectiveness of the E-PLAYS intervention when delivered by SLTs and teaching assistants by comparison to usual care for children with social communication impairments.

To assess feasibility, we examined recruitment of children through the Speech and Language Services of the NHS, the acceptability of E-PLAYS to the children and teaching assistants using it, methods of determining intervention fidelity and delivery and the suitability of outcome measures and cost-effectiveness measures. Important lessons were learned that will be applied to any follow-on trial.

### Recruitment and retention

Our original target was to recruit 70 children over a period of 3 months [56, 71]. We actually recruited 50 children in 3.5 months although the recruitment period had to be delayed. For a full trial, increasing the number of children to be recruited could be addressed straightforwardly by simply recruiting more NHS trusts to the study. Although 70 children had been our original target, there are no set guidelines for participant numbers for feasibility trials; sample sizes of between 24 and 70 have been recommended to allow for the reliable estimation of the standard deviation for use in the sample size calculation of a future fully powered trial [56, 72] and 50 was in fact sufficient for this purpose. Importantly, our E-PLAYS intervention has already indicated a signal of efficacy in pilot studies [29, 44].

The time period within which to recruit children and the rate of recruitment are more complex issues as they are constrained by the necessity of operating within the school year. We delayed the recruitment period as far as possible to allow SLTs time to examine their caseloads and approach schools. Even with this delay, however, we still did not manage to recruit our target of 70 children. We were obliged to bring recruitment to a close in order to begin intervention delivery within the school year. A major factor causing recruitment delays was that SLTs often did not know which children were to be on the caseload until well into the school year. However, for a full trial, recruitment could be more easily managed in waves over a 2- or 3-year period; once a school and SLT are part of the study, children from subsequent school years can be recruited relatively easily. E-PLAYS was highly appealing, and thus, schools and SLTs were generally keen to participate. We recruited 50 children from a single NHS trust; our sample size calculation suggests that we would need 356 children and 71 NHS SLTs for a full trial. This would necessitate the recruitment of around 6–7 NHS trusts, which would appear feasible.

An important feasibility question concerned recruitment of the typically developing peer children to partner the focal children and the willingness of parents to allow their participation. We found that 100% of parents gave consent for these children to take part. However, concerns have been expressed elsewhere that this kind of participation could have negative consequences for typically-developing children [73, 74] as it may take up their own educational time for the benefit of other children. Locke et al.’s (2012) longitudinal study [75] did not report any adverse outcomes for typically developing children as a result of participating in a peer-mediated intervention, and the burden of our E-PLAYS intervention is relatively light (4 × 15-min sessions). E-PLAYS aims to provide a positive educational experience for typically-developing children as well as those with communication impairments and teaching assistants reported that they enjoyed it just as much.

Retention was satisfactory; 70% retention is considered the minimum for inclusion in Cochrane Reviews [76]. Participant burden on the children did not appear to be onerous which seems to be borne out by children’s willingness when approached by research assistants. Teacher and parent responses were slightly lower than 70% at 40 weeks. Although the questionnaire burden on teachers was relatively light (15 min per child), we found that this was a stressed occupational group with high staff turnover. As we were relying on schools to pass questionnaires on, this also impacted the response rate from parents. For a full trial, we will explore possibilities for incentives for schools. This has worked successfully in other large-scale school-based NIHR trials.

### Acceptability

E-PLAYS was highly acceptable, with SLTs, teaching assistants and children all rating it very positively. It is likely that this reflects the high level of intervention development, testing and stakeholder involvement in the pilot study that preceded the feasibility study [29, 44].

The questionnaires to SLTs and teaching assistants concerning training and the manual elicited favourable responses, suggesting that we had been able to develop accessible instruction material. All reported that the computer game was easy to run. There were few issues around acceptability. Feedback overwhelmingly consisted of requests for a higher standard of graphics and additional games features which will be considered for future versions.

A further reason for the popularity of E-PLAYS was that it had been designed to fit within normal school and SLT working patterns. Thus, additional staff and resources were not required from schools and NHS SLTs taking part.

Tolmie et al. (2010) [24] found that collaborative work led to improved student relations. This is a particularly important consideration for children with special educational needs who are generally less liked and accepted by their peers; Pinto et al. (2019) [31] found that the level of *meaningful interaction* with peers was the strongest predictor of acceptability for these children. Collaborative computer games are generally enjoyable interactions but also *meaningful* in this instance as the children in E-PLAYS are directed and obliged to collaborate to succeed in the game. It is possible, therefore, that E-PLAYS may have an impact on children’s peer relations and this is one of the measures we propose to include in a future trial. Teaching assistants reported that sessions with peer classmates were particularly popular, with most suggesting that these should be increased in number and duration.

### Treatment fidelity and delivery

Should E-PLAYS ultimately become nationally available and distributed, it is important that SLTs and teaching assistants can use it autonomously. For maximum fidelity, E-PLAYS was web-based and manualised with the content for each session specified step-by-step. The manual was generally well-liked and sessions were delivered without major problems thereby showing promise for good fidelity in a national roll-out. Delays tended to occur around the commencement of the intervention, chiefly due to staff shortages. However, once schools began with E-PLAYS, they were keen to continue.

We were able to gain precise assessment of treatment delivery through automatic recording by the E-PLAYS software. This recording was supplemented with live observations of the teaching assistants delivering E-PLAYS. These two methods confirmed that delivery was acceptable in terms of number and length of sessions.

### Suitability of outcome measures

Pragmatic language skills are notoriously difficult to measure; the problem being that they manifest themselves only during dynamic social interaction, thus rendering testing with standardised questionnaires largely unachievable [1]. Furthermore, adult (particularly parent) report for conditions relating to autism and social communication are thought to be particularly susceptible to placebo effects [77, 78]. In spite of this, the majority of social communication literature is based on non-blinded parent-, teacher- or clinician-report [79]. It was one of our chief objectives to address this limitation and use effective measures administered by independent, blinded outcome assessors. We therefore used the TPS and CELF-5 subscales administered by research assistants. For consistency with other studies and to provide more global measures we also included reports from teachers and parents.

A key outcome of language and communication skill-learning is measuring subsequent use of the skill in contexts other than the one in which it was learned; skills can fail to generalise to novel contexts. Wieckowski and White’s (2017) [4] systematic review of technological interventions for social communication impairments reports that this is rarely assessed. To measure generalisation we included the CCT, previously devised by the authors.

The suitability of our measures was indicated by the good response and retention rates. Also, completion of the measures was excellent with very few missing items; almost all were analysable (see Fig. 1). Comparison of scores with norms suggests that these measures will detect changes as a result of intervention in a future full trial. We were able to preserve blinding for the research assistant-administered measures and for the CCT to successfully train research assistants to high levels of inter-rater reliability.

### Health economic measures and cost-effectiveness

At present, neither of the instruments tested (ED-5D-Y proxy-1 and PedsQL) can be used to calculate QALYs that are required for a utility analysis, nor is there an alternative that can be used for this age range. However, EuroQol are currently looking to establish a value set for children and this may be available in the near future. On this basis, we would plan to include both the EQ-5D-Y proxy-1 and PedsQL in a full trial to ensure the best representation of health-related quality of life with the view of calculating QALYs if possible. Completion rates for EQ-5D proxy-1 and PedsQl were very similar to each other but PedsQL provides more information on social and emotional functioning.

As the feasibility study was conducted with a small number of participants in a geographically small area, the low uptake of resources may reflect low levels of availability within this area. This is at odds with previous research; health economic evaluations have been called for in this field as it has been shown that healthcare costs are 36% higher for children with language disorders aged 4–5 years [80]. In a full-scale trial, we may observe higher uptake owing to greater availability resources at a national level. This would also allow us to examine the effect of the intervention from a societal perspective.

### Strengths and limitations

The low-cost, computer-based nature of E-PLAYS makes it highly suitable for national distribution. E-PLAYS was designed ultimately to be widely shared and we have used existing, easily updated technology which is available within the NHS and in primary schools. A vast number of commercially-available games are targeted at parents of children with autism spectrum disorders and other children with social communication impairments [81]. Reviewers [36] of the computer game literature have urgently called for large-scale studies to ensure that exploitation of the opportunities available with these new technologies is founded on a sound evidence-base to benefit the children to whom they are marketed.

Particular strengths of this study are that we have included independent, blinded measures and also a measure of generalisation of the skills taught. Through the use of technology, we were also able measure delivery of the intervention sessions precisely; other studies have been obliged to depend on less reliable methods such as teacher reports and diary methods.

Fidelity was further assessed via direct live observation of teaching assistants delivering some of the E-PLAYS sessions. Teaching assistants knew that they were being observed and this may have impacted on their behaviour when delivering these particular sessions. Possible alternatives could involve video-recording every session and then selecting random sessions for fidelity assessment. This would have the advantage that teaching assistants would not know which sessions were to be observed and could not alter their behaviour accordingly. However, this would have considerable resource implications for a large-scale study requiring either that a research assistant attend every session to record it or that teaching assistants were asked to make all the recordings themselves. A further drawback would be that teaching assistants and children would only use E-PLAYS with constant observation, thereby possibly not reflecting natural usage of the intervention which could impact on outcomes. A limitation of our study concerns the demographic makeup of our sample. Demographics were taken at the 40-week data collection point thereby probably resulting in a lower response rate than had we collected this information at baseline. We acknowledge that this is a shortcoming of our study and that the value of this data is correspondingly reduced. Responses came primarily from white British participants with an above-average level of education; this did not reflect the highly diverse areas of London in which the study took place. However, our finding is consistent with those of Safer-Lichtenstein et al.’s systematic review (2019) [82] who report that study samples for children with autism generally lack diversity, with an overrepresentation of participants who are male, White, and from upper-middle class backgrounds. This is particularly concerning in view of the fact that children from lower socio-economic backgrounds are less likely to receive diagnoses and support from health and education services for autism or language disorders [10, 12]. Sample composition is an important consideration, particularly if we fail to include groups that are often under-represented. We will explore means of recruiting a more diverse sample for a future RCT.

## Conclusion

Based on the outcomes of our study, a full-scale trial appears feasible and warranted to assess the effectiveness of E-PLAYS for use by the NHS and schools. Participants in this study (focal children, teaching assistants, SLTs) enjoyed using E-PLAYS; overall acceptability was high. Few concerns were reported regarding participation in a future trial. However, strategies to improve recruitment rate and retention and to recruit a more diverse sample would need to be adopted. Despite some limitations, this feasibility study prepares the ground for one of the first interventions for children with social communication impairments to harness the potential of computer technology. A full-scale trial would result in making a valuable resource available as well as advancing our knowledge of how to design technological interventions tailored for this group of children.

## Trial status

Ethical approval was obtained on 4th December 2017 (Cambridge Central Research Ethics Committee, REC ref: 17/EE/0320, IRAS Project ID: 227864). The study opened to recruitment on 1st January 2018 and completed recruitment on 30th March 2018.

## Data Availability

The datasets generated during and/or analysed during the current study have been stored in non-publically available repositories within the Universities of York and Bedfordshire. Anonymised data may be accessed and analysed by members of the project team and with researchers collaborating with members of the project team on the analysis of these data. Consent from participants was not sought for sharing raw data publicly. Therefore, external researchers seeking to access the data for use in future projects can do so via any reasonable request to the Chief Investigator Suzanne Murphy (or her delegate), and projects using the data must have been approved in accordance with contemporary UK ethical and regulatory processes pertaining to the release of anonymised data under these circumstances.

## Abbreviations

CCC-2: Children’s Communication Checklist-2
CCT: Collaborative construction task
CELF-5: Clinical Evaluation of Language Fundamentals-5
DSM-V: Diagnostic and Statistical Manual of Mental Disorders
E-PLAYS: Enhancing Pragmatic Language skills for Young children with Social communication impairments
EQ-5D: EuroQol 5-Dimension scale
MRC: Medical Research Council
NELFT: NorthEast London NHS Foundation Trust
NHS: National Health Service
PedsQL: Paediatric Quality of Life inventory
SDQ: Strengths and Difficulties Questionnaire
SLT: Speech and language therapist
TPS: Test of Pragmatic Skills

## Footnotes

1 95% bias corrected bootstrap confidence intervals.

2 95% confidence intervals for correlations based on Fisher’s z-transformation.
